# Intergenerational transmission of attachment patterns in families of adolescents with non-suicidal self-injury: the mediating role of child insecure attachment

**DOI:** 10.1186/s12888-026-08385-3

**Published:** 2026-07-15

**Authors:** Ya-Qiong Li, Jia-Ting Li, Si Mi, Ying-Zhe Zhao, Shan Wang, Jing Liu

**Affiliations:** 1 Beijing Key Laboratory of Intelligent Drug Research and Development for Mental Disorders, National Clinical Research Center for Mental Disorders, National Center for Mental Disorders, Beijing Anding Hospital, Capital Medical University, No. 5, Ankang Hutong, Deshengmenwai Street, Xicheng District, Beijing, 100088 China; 2 Independent Scholar, Beijing, 100020 China

**Keywords:** Adolescents, Attachment, Intergenerational transmission, Non-suicidal self-injury (NSSI), Parent-child relations, Structural equation modeling

## Abstract

**Objective:**

The objective of this study was to evaluate the direct and indirect associations between parental insecure attachment and non-suicidal self-injury (NSSI) in adolescents, with particular emphasis on the mediating role of insecure attachment in the child and the differential contributions of paternal and maternal attachment patterns.

**Methods:**

A cross-sectional design was employed involving 74 adolescents aged 10–19 years and their parents. Participants were categorized into three groups: adolescents with NSSI (NSSI group), adolescents diagnosed with mood disorders without NSSI (clinical control group), and healthy controls. Attachment patterns were assessed using the Experiences in Close Relationships Inventory. Mediation effects were examined using structural equation modeling.

**Results:**

Insecure attachment in the child was a significant predictor of NSSI in adolescents (β = 0.823, *p* = 0.003). Neither paternal nor maternal insecure attachment demonstrated a direct association with NSSI. However, insecure attachment in fathers was significantly associated with insecure attachment in the child (β = 0.852, *p* = 0.043). A statistically significant indirect effect was observed between paternal insecure attachment and adolescent NSSI through insecure attachment in the child (β = 0.701, *p* = 0.047). The corresponding mediation pathway involving maternal insecure attachment was not statistically significant.

**Conclusions:**

Insecure attachment in the child served as a key mediating factor in the association between paternal insecure attachment and NSSI. These findings suggest that paternal attachment patterns may have a distinct influence on the development of attachment insecurity in adolescents. Preventive and therapeutic interventions may benefit from addressing attachment security in both parents and children, with particular consideration given to paternal attachment within intergenerational transmission processes.

**Clinical trial number:**

Not applicable.

## Introduction

Non-suicidal self-injury (NSSI) constitutes a significant public health concern among adolescents and is defined as the intentional, direct destruction of body tissue in the absence of suicidal intent. This behavior is associated with substantial emotional distress and an increased risk of subsequent suicidal behaviors [1–4]. Although the prevalence of NSSI among adolescents varies across populations, consistently high rates have been reported in both international studies and research conducted in China, underscoring the need to clarify underlying mechanisms to inform the development of targeted prevention strategies [2, 5, 6].

Attachment theory provides a conceptual framework for understanding the development of NSSI in adolescents within the family context. Secure attachment, established through consistent and supportive caregiver–child interactions, is associated with healthy emotional development and adaptive coping capacities. In contrast, insecure attachment patterns, including avoidant, anxious, and fearful styles, are frequently associated with a range of maladaptive behaviors, including self-injurious behavior, due to difficulties in regulating negative emotions and managing stress [7–10]. Emerging research has suggested that mentalization and social cognition may represent important mechanisms linking insecure attachment to NSSI [11]. Mentalization refers to the capacity to understand one’s own and others’ thoughts, feelings, and intentions, and is considered essential for adaptive emotional regulation and interpersonal functioning. Adolescents with insecure attachment patterns have been shown to exhibit reduced mentalizing capacity and greater difficulties interpreting emotional experiences, which may increase vulnerability to maladaptive coping responses under conditions of psychological distress. From this perspective, NSSI may function as a behavioral response to unprocessed emotional pain, psychache, or emotional detachment when more adaptive strategies for understanding and regulating affect are unavailable [12]. Consequently, deficits in mentalization and social cognition may constitute important intermediate pathways through which attachment insecurity contributes to the emergence of self-injurious behaviors during adolescence [13, 14]. Accordingly, parental insecure attachment may indirectly elevate the risk of NSSI during adolescence by contributing to the development of insecure attachment in the child and associated difficulties in emotional regulation, interpersonal functioning, and mentalization [8, 15].

Although a substantial body of research has consistently demonstrated an association between insecure attachment in adolescents and NSSI [9, 15–17], much of this literature has focused primarily on individual-level factors and has given limited consideration to broader family dynamics. In particular, the distinct contribution of paternal attachment patterns has received relatively limited attention, despite growing evidence indicating that fathers play a significant role in the emotional and social development of adolescents [18–22]. As insecure attachment in both mothers and fathers may influence the risk of NSSI during adolescence, potentially through the development of insecure attachment in the child, the examination of both paternal and maternal attachment orientations offers a more comprehensive understanding of intergenerational attachment-related processes [20, 23, 24].

Accordingly, the present study examined the direct and indirect associations between parental insecure attachment and NSSI in adolescents, with insecure attachment in the child conceptualized as a central mediating factor. Specifically, paternal and maternal insecure attachment patterns were evaluated in relation to their respective contributions to insecure attachment in adolescents and the subsequent likelihood of NSSI. By delineating the potentially distinct roles of fathers and mothers, this investigation aimed to enhance understanding of the intergenerational transmission of insecure attachment patterns and their manifestation as self-injurious behavior during adolescence. Furthermore, the findings emphasized the importance of concurrently addressing parental attachment orientations and insecure attachment in adolescents, thereby informing the development of comprehensive, attachment-focused interventions intended to prevent and reduce NSSI among adolescents.

## Method

### Participants and recruitment

A cross-sectional study design was utilized and included adolescents aged 10–19 years and their parents. Participants were allocated into three groups: adolescents with NSSI (NSSI group), adolescents diagnosed with mood disorders without a history of NSSI (clinical control group), and healthy adolescents without a current or past psychiatric diagnosis (healthy control group). Adolescents in the NSSI and clinical control groups were recruited from outpatient and inpatient psychiatric services at a specialized psychiatric hospital. Mood disorder diagnoses were established according to DSM-5 criteria through routine clinical assessment conducted by experienced psychiatrists. Outpatient participants were referred by treating psychiatrists based on eligibility criteria, whereas inpatient diagnoses had been confirmed through the hospital’s standard diagnostic review procedures involving attending and senior psychiatrists. Eligibility for the NSSI group was determined based on clinical documentation of at least one episode of non-suicidal self-injury within the preceding 12 months, as recorded in medical records and confirmed by the treating psychiatrist during recruitment. The healthy control group was recruited through voluntary community-based online advertisements.

Eligibility criteria for the NSSI group included a diagnosis of a mood disorder based on Diagnostic and Statistical Manual of Mental Disorders, Fifth Edition diagnostic criteria through routine psychiatric evaluation, a documented history of NSSI within the preceding 12 months based on clinical records, and informed consent by the adolescent and at least one parent.

Exclusion criteria for all groups included the presence of intellectual disability, severe medical conditions, or receiving electroconvulsive therapy within the previous three months. The same age range and diagnostic criteria were applied to the clinical control group, with the additional requirement of no lifetime history of NSSI. Adolescents in the healthy control group were screened to confirm the absence of psychiatric diagnoses and the absence of a family history of psychiatric disorders.

Parents across all study groups were required to have completed at least a primary school level of education and to have no history of severe psychiatric disorders or intellectual disability. Written informed consent for study participation was obtained from both adolescents and their parents.

### Measures

#### Demographics and background information

Participants completed a demographic questionnaire that collected information on age, sex, educational attainment, family structure, and perceived economic pressure. Economic pressure was evaluated using a four-item scale assessing clothing, food, housing, and transportation needs, with responses rated on a 5-point Likert scale. Higher total scores reflected greater levels of perceived economic pressure.

#### Attachment

Attachment patterns in adolescents and their parents were assessed using the Experiences in Close Relationships Inventory (ECR), a validated self-report instrument that evaluates two core dimensions of attachment: attachment-related anxiety and attachment-related avoidance. Based on established scoring procedures, participants were categorized as having secure or insecure attachment patterns, including avoidant, anxious, or fearful attachment styles. For the assessment of adolescent attachment to parents, the ECR was adapted by substituting the term “partner” with “parent” throughout the questionnaire.

#### Parenting style

Parental rearing styles were assessed using the Simplified Parenting Style Scale, revised by Jiang et al. [25]. The scale includes two parallel subscales for fathers and mothers, each consisting of three dimensions: rejection, emotional warmth, and overprotection, with 21 items per subscale. Participants rated items on a 4-point Likert scale, with higher scores indicating stronger endorsement of the corresponding parenting style. In the present study, father warmth, mother warmth, father rejection, and mother rejection were treated as observed variables in the structural equation model. These variables were derived from the corresponding the Simplified Parenting Style Scale subscale scores. The scale has demonstrated good reliability and validity in Chinese populations [25].

### Procedures

Data collection was conducted between December 2021 and December 2022. Adolescents and their parents independently completed self-report questionnaires in a quiet, designated setting under the supervision of trained research assistants. All measures were administered during a single assessment session lasting approximately 60–90 min. Research assistants provided clarification on questionnaire items when necessary and responded to participant inquiries. To reduce the potential for response bias, confidentiality was emphasized, and neutral wording was employed across all questionnaire materials.

### Statistical analyses

Statistical analyses were conducted using Python (version 3.10) and R (version 4.3.0). Data preprocessing, descriptive analyses, and basic inferential statistical procedures were performed in Python using the pandas, numpy, scipy, statsmodels, and seaborn packages. Structural equation modeling (SEM) was conducted in R using the lavaan package.

Continuous variables were summarized using means and standard deviations (mean ± SD), whereas categorical variables were presented as frequencies and percentages. The distributional normality of continuous variables was evaluated using the Shapiro-Wilk test, and homogeneity of variances was assessed using Levene’s test. Between-group comparisons were conducted using one-way analysis of variance (ANOVA) when assumptions of normality and homoscedasticity were satisfied, while the Kruskal-Wallis test was applied when these assumptions were not met. Categorical variables were compared using chi-squared tests.

Logistic regression models were employed to examine associations between insecure attachment patterns in the child, father, and mother and NSSI status, with results reported as regression coefficients, *p*-values, and odds ratios. In addition, exploratory regression analyses were conducted to assess associations among parental insecure attachment, parenting styles, insecure attachment in the child, and NSSI.

To further examine the associations and pathways linking parental insecure attachment, insecure attachment in the child, and NSSI, SEM was conducted using R. The SEM incorporated both direct pathways, including the effects of parental insecure attachment, age, and sex on insecure attachment in the child and NSSI, as well as indirect pathways evaluating the effect of parental insecure attachment on NSSI mediated by insecure attachment in the child. Model estimation was conducted using the weighted least squares mean and variance-adjusted estimator, which is appropriate for models with categorical outcome variables. Standardized regression coefficients, corresponding *p*-values, and 95% confidence intervals were reported for all specified pathways.

All statistical tests were two-sided, and a significance threshold of α = 0.05 was applied across all analyses.

## Results

The study sample consisted of 74 adolescent–parent triads, including 40 adolescents diagnosed with mood disorders and a history of NSSI, 18 adolescents diagnosed with mood disorders without a history of NSSI, and 16 healthy adolescents without psychiatric diagnoses.

Comparative analyses of demographic and socioeconomic characteristics across the three groups are presented in Table 1. Group differences in continuous variables were evaluated using one-way analysis of variance, whereas categorical variables were assessed using chi-square tests. No statistically significant differences were observed for any of the variables examined, indicating the comparability of the groups with respect to demographic and socioeconomic characteristics.

**Table 1:** Comparisons of demographic and socioeconomic characteristics across study groups

Variable	Statistic	*P*-Value
Adolescent’s age^1^	0.340	0.713
Adolescent’s gender	3.268	0.195
Adolescent’s nationality	4.712	0.095
Father’s age^1^	0.033	0.968
Father’s education level	4.849	0.901
Father’s occupation	9.910	0.769
Mother’s age^1^	0.031	0.970
Mother’s education level	8.071	0.622
Mother’s occupation	9.171	0.820
Per capita monthly household income	9.053	0.338

^1^ ANOVA

### Attachment styles

Comparisons of attachment-related anxiety and avoidance scores among adolescents diagnosed with mood disorders with NSSI, mood disorders without NSSI, and healthy controls are summarized in Table 2. Statistically significant group differences were observed for avoidance scores in adolescents, with both mood disorder groups demonstrating higher levels of attachment-related avoidance compared to the healthy control group (Kruskal-Wallis statistic = 24.65, *p* < 0.001). In contrast, attachment-related anxiety scores in adolescents did not differ significantly across groups; however, a non-significant trend toward higher anxiety scores was noted in the NSSI group compared to the healthy control group (Kruskal-Wallis statistic = 5.54, *p* = 0.06).

**Table 2:** Comparisons of attachment-related anxiety and avoidance scores among adolescents across study groups

Variable	HC	MD	NSSI	Statistic	*P*-Value
child_avoidance	2.7 ± 0.53	4.6 ± 0.88	4.4 ± 1.23	24.65	0.00
child_anxiety	2.8 ± 0.56	3.1 ± 1.00	3.5 ± 1.21	5.54	0.06
father_avoidance^1^	2.5 ± 0.67	3.0 ± 0.82	3.2 ± 0.68	5.12	0.01
father_anxiety^1^	2.9 ± 0.70	3.3 ± 1.00	3.4 ± 0.91	1.76	0.18
mother_avoidance^1^	2.5 ± 0.74	3.0 ± 0.92	2.7 ± 0.95	1.23	0.30
mother_anxiety^1^	2.9 ± 0.64	3.3 ± 0.82	2.9 ± 0.77	1.62	0.20

^1^ ANOVA

With respect to parental attachment patterns, significant group differences were identified in attachment-related avoidance scores among fathers, with higher scores observed in the mood disorder groups compared to the healthy control group (ANOVA statistic = 5.12, *p* = 0.01). No statistically significant group differences were detected for attachment-related anxiety scores among fathers (ANOVA statistic = 1.76, *p* = 0.18), attachment-related avoidance scores among mothers (ANOVA statistic = 1.23, *p* = 0.30), or attachment-related anxiety scores among mothers (ANOVA statistic = 1.62, *p* = 0.20).

The findings suggest that attachment-related avoidance, particularly among adolescents and fathers, was more pronounced in adolescents diagnosed with mood disorders, whereas attachment-related anxiety did not differ significantly across the study groups.

Table 3 summarizes the distribution of attachment patterns (secure, anxious, avoidant, and fearful) among adolescents, fathers, and mothers across the healthy controls (HC), mood disorder without NSSI (MD), and mood disorder with NSSI (NSSI) groups. Among adolescents, statistically significant differences were observed in the distribution of attachment patterns across groups (χ² = 44.36, *p* < 0.001). Secure attachment type was predominant in the HC group (100.0%), whereas avoidant (40.0%) and fearful (32.5%) attachment patterns were more frequently observed in the NSSI group. In the MD group, avoidant attachment was the most prevalent pattern (61.1%). In contrast, the distributions of attachment patterns among fathers and mothers demonstrated less variability across groups, with no statistically significant group differences detected. Secure attachment was the most common pattern among both fathers and mothers across all groups, with comparatively smaller proportions of anxious, avoidant, and fearful attachment patterns. Overall, these results suggest that adolescents with mood disorders and NSSI were more likely to exhibit insecure attachment patterns than healthy controls, whereas parental attachment patterns appeared relatively consistent across groups.

**Table 3:** Distribution of attachment patterns among adolescents, fathers, and mothers across study groups

Attachment Type		HC	MD	NSSI	Chi2	*P*-Value
Child	Secure	16 (100.0%)	3 (16.7%)	6 (15.0%)	44.36	0.00
	Anxious	0 (0.0%)	2 (11.1%)	5 (12.5%)		
	Avoidant	0 (0.0%)	11 (61.1%)	16 (40.0%)		
	Fearful	0 (0.0%)	2 (11.1%)	13 (32.5%)		
Father	Secure	14 (87.5%)	11 (61.1%)	26 (65.0%)	5.3	0.506
	Anxious	2 (12.5%)	3 (16.7%)	9 (22.5%)		
	Avoidant	0 (0.0%)	2 (11.1%)	3 (7.5%)		
	Fearful	0 (0.0%)	2 (11.1%)	2 (5.0%)		
Mother	Secure	16 (100.0%)	14 (77.8%)	31 (77.5%)	7.66	0.264
	Anxious	0 (0.0%)	3 (16.7%)	4 (10.0%)		
	Avoidant	0 (0.0%)	0 (0.0%)	4 (10.0%)		
	Fearful	0 (0.0%)	1 (5.6%)	1 (2.5%)		

**Fig. 1:**
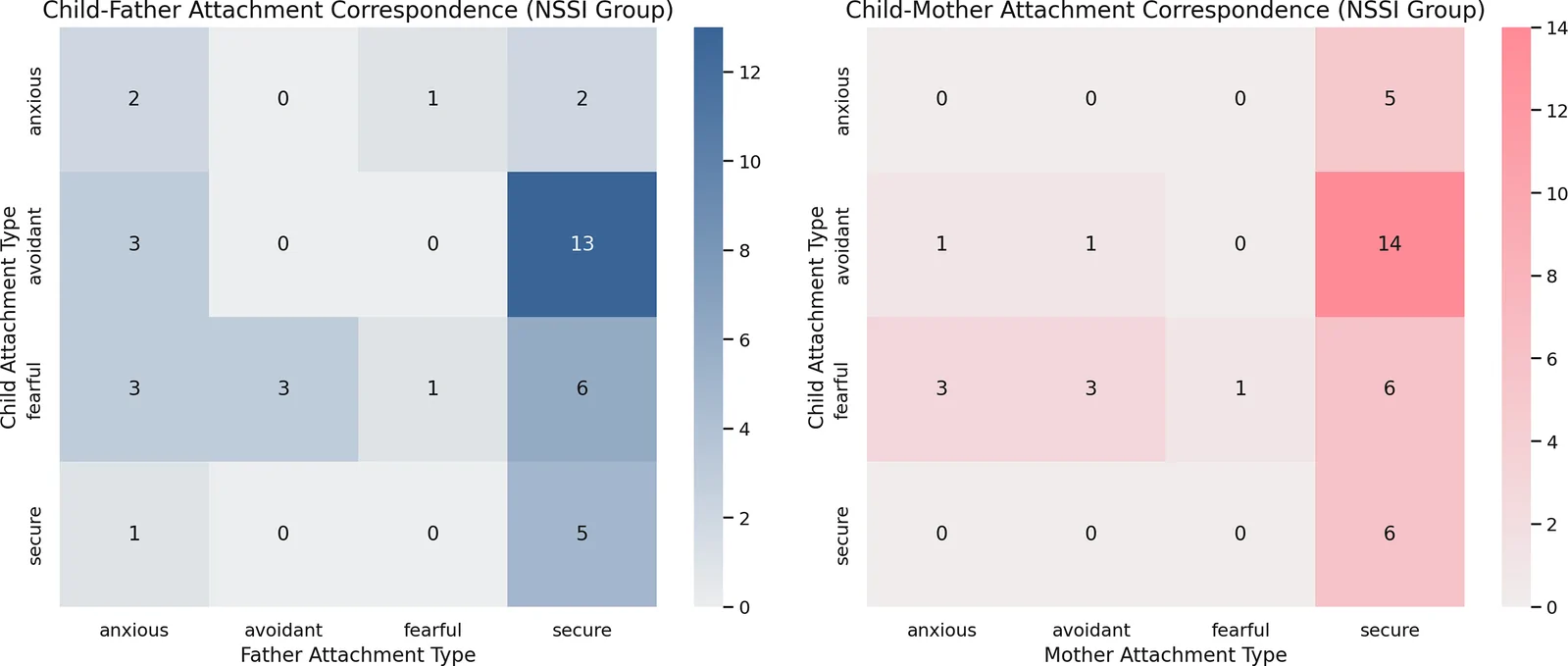
Correspondence between attachment patterns in adolescents and fathers or mothers within the NSSI group

Correspondence patterns between attachment classifications in adolescents and their parents within the NSSI group are illustrated in Fig. 1. For adolescent-father attachment correspondence, adolescents characterized by fearful attachment most frequently corresponded to fathers classified as having secure attachment (13 cases). This pattern indicated that adolescents exhibiting insecure attachment patterns often had fathers with a stable secure attachment classification. Other adolescent attachment patterns, including avoidant and anxious attachment, demonstrated more heterogeneous distributions across paternal attachment classifications, with no distinct correspondence pattern observed.

Regarding adolescent-mother attachment correspondence, adolescents classified as having secure attachment most frequently corresponded to mothers with secure attachment (14 cases). In addition, mothers classified as having fearful attachment more frequently corresponded to adolescents with fearful attachment (3 cases). This pattern suggested that maternal insecure attachment, in certain instances, aligned with insecure attachment in adolescents, particularly fearful attachment.

Overall, adolescents in the NSSI group predominantly demonstrated insecure attachment patterns, particularly avoidant and fearful attachment, whereas parents, particularly fathers, were more frequently classified as having secure attachment patterns. This divergence underscored the complexity of parent-child attachment relationships and suggested that family dynamics and adolescent-specific psychological factors may contribute to the development and expression of attachment patterns during adolescence.

### SEM analysis

**Fig. 2:**
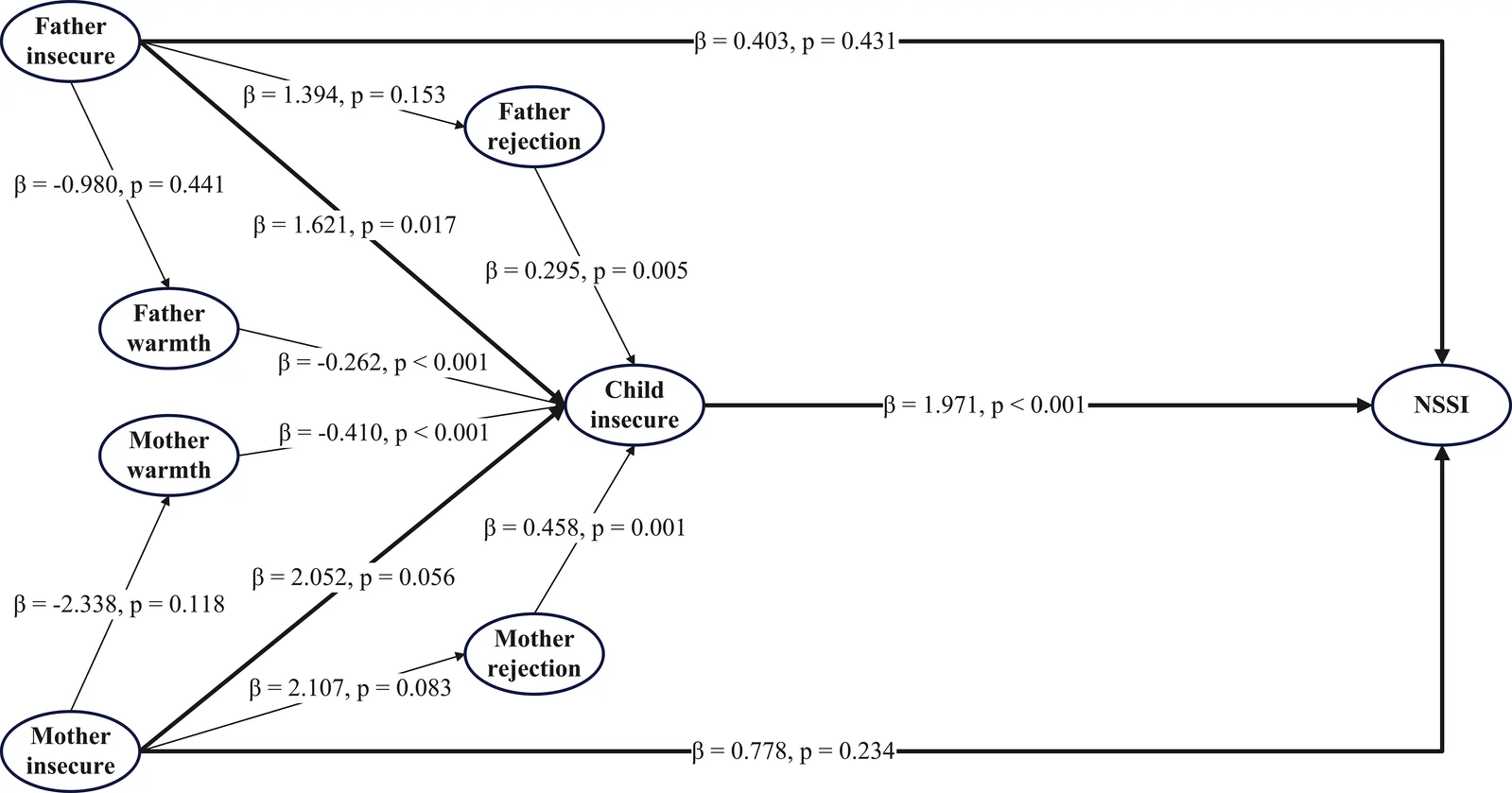
Structural equation model illustrating direct pathways among parental insecure attachment, insecure attachment in adolescents, and NSSI

The SEM analysis revealed several statistically significant findings concerning the associations among parental insecure attachment, insecure attachment in the child, and NSSI. As illustrated in Fig. 2, the path diagram depicts the specified structural relationships and indicates that the association between paternal insecure attachment and NSSI was fully mediated by insecure attachment in the child, with no evidence of a significant direct pathway.

### Direct effects

The direct effects of parental insecure attachment, age, and sex on insecure attachment in the child and NSSI are summarized in Table 4, which presents both statistically significant and non-significant pathways along with the corresponding regression coefficients and *p*-values. Insecure attachment in fathers significantly predicted insecure attachment in the child (β = 0.852, SE = 0.420, z = 2.027, *p* = 0.043), indicating that higher levels of paternal insecure attachment were associated with increased insecure attachment in adolescents. Insecure attachment in mothers also demonstrated a positive association with insecure attachment in the child; however, this relationship did not reach statistical significance (β = 1.061, SE = 0.642, z = 1.653, *p* = 0.098).

**Table 4:** Results of regression path analyses examining direct effects in the structural equation model

Dependent Variable	Independent Variable	Coefficient (B)	SE	z-Value	*p*-Value	95% CI	Beta (Std. All)
child_insecure	father_insecure	0.852	0.420	2.027	0.043	[0.028, 1.676]	0.331
child_insecure	mother_insecure	1.061	0.642	1.653	0.098	[-0.197, 2.319]	0.339
child_insecure	age	-0.013	0.078	-0.165	0.869	[-0.165, 0.139]	-0.021
child_insecure	gender	-0.419	0.436	-0.961	0.337	[-1.273, 0.435]	-0.138
NSSI	father_insecure	-0.452	0.417	-1.085	0.278	[-1.269, 0.365]	-0.158
NSSI	mother_insecure	-0.282	0.603	-0.468	0.640	[-1.464, 0.900]	-0.081
NSSI	child_insecure	0.823	0.276	2.984	0.003	[0.282, 1.364]	0.739
NSSI	age	0.012	0.095	0.130	0.896	[-0.173, 0.198]	0.018
NSSI	gender	0.849	0.487	1.743	0.081	[-0.106, 1.803]	0.250

Insecure attachment in the child was a significant predictor of NSSI (β = 0.823, SE = 0.276, z = 2.984, *p* = 0.003), underscoring its central role in the occurrence of self-injurious behavior. In contrast, neither insecure attachment in fathers (β = -0.452, *p* = 0.278) nor insecure attachment in mothers (β = -0.282, *p* = 0.640) demonstrated a significant direct association with NSSI. Additionally, the effects of age and sex on insecure attachment in the child and NSSI were not statistically significant, with the exception of a marginal association between sex and NSSI (β = 0.849, SE = 0.487, z = 1.743, *p* = 0.081).

### Indirect effects

The indirect associations between parental insecure attachment and NSSI through insecure attachment in the child are presented in Table 5. A statistically significant indirect effect was observed for insecure attachment in fathers on NSSI through insecure attachment in the child (β = 0.701, SE = 0.352, z = 1.990, *p* = 0.047), indicating a mediating role of insecure attachment in the child within this relationship. In contrast, although insecure attachment in mothers also demonstrated an indirect association with NSSI through insecure attachment in the child, this effect did not reach statistical significance (β = 0.873, SE = 0.574, z = 1.521, *p* = 0.128).

**Table 5:** Results of indirect effects analyses examining mediation pathways in the structural equation model

Indirect Effect	Pathway	Coefficient (β)	SE	z-Value	*p*-Value	95% CI	Beta (Std. All)
indirect_father	father → child → NSSI	0.701	0.352	1.990	0.047	[0.011, 1.392]	0.244
indirect_mother	mother → child → NSSI	0.873	0.574	1.521	0.128	[-0.252, 1.999]	0.250

## Discussion

The present study sought to clarify the intergenerational transmission of attachment patterns and their association with NSSI during adolescence, with particular emphasis on the mediating role of insecure attachment in the child. The findings supported the hypothesis that insecure attachment in adolescents constituted a primary pathway through which parental insecure attachment patterns were associated with NSSI. Although insecure attachment in parents did not demonstrate a direct association with NSSI, insecure attachment in adolescents was significantly associated with an increased likelihood of self-injurious behavior. Collectively, these findings highlighted the importance of simultaneously considering attachment-related factors at both the parental and adolescent levels when examining developmental pathways underlying NSSI.

In accordance with attachment theory, insecure attachment in adolescents may be indicative of early relational experiences shaped through interactions with parents [26–29]. This interpretation is consistent with established theoretical models proposing that insecure attachment patterns formed during sensitive developmental periods may contribute to maladaptive emotion regulation strategies, including self-injurious behaviors during adolescence [30–32]. By demonstrating that parental insecure attachment was indirectly associated with NSSI through insecure attachment in adolescents, these findings underscored the central role of attachment insecurity in the child within intergenerational transmission processes.

With respect to paternal and maternal insecure attachment, both were associated with attachment security in adolescents, although their contributions appeared to differ in magnitude and pattern. Despite the historical emphasis on maternal influences in attachment research, the findings indicated that paternal attachment patterns also played a significant role in the intergenerational transmission of insecure attachment. An unexpected finding was that adolescents classified as having fearful attachment were frequently associated with secure paternal attachment. Although this result appears counterintuitive in relation to classical intergenerational transmission models, it may reflect more complex family dynamics and boundary conditions of attachment transmission. One possible explanation is that secure paternal attachment may not uniformly translate into reduced attachment insecurity in adolescents, particularly when other risk factors (e.g. maternal attachment insecurity, family emotional climate, or individual vulnerability) are present. In addition, fathers may contribute more strongly to autonomy development than emotional regulation, and a secure paternal attachment may therefore coexist with emotional insecurity in adolescents if emotional needs are not consistently met by other caregivers. It is also possible that this pattern reflects developmental differences in father–adolescent relationships or limitations related to sample size and group classification. Although insecure attachment in fathers and mothers did not independently predict NSSI, their associations with insecure attachment in adolescents highlighted the importance of involving both parents in preventive and therapeutic interventions aimed at reducing the risk of self-injurious behavior [33, 34].

Developmental attachment research has emphasized that mothers and fathers may contribute differently to the development of psychological security across the lifespan. Maternal attachment representations are more strongly associated with affect regulation processes in children, including emotional expression, attention regulation, and comfort seeking, whereas paternal attachment has been more closely linked to exploratory behavior, autonomy development, and self-confidence. Grossmann et al. [35] further highlighted the role of paternal play sensitivity in shaping long-term psychological security. The present findings may reflect differential pathways through which paternal attachment influences adolescent outcomes. Paternal secure attachment may contribute more strongly to autonomy-related dimensions of development rather than directly buffering emotional dysregulation underlying NSSI. This may explain the observed complexity in the association between paternal attachment security and adolescent fearful attachment patterns, suggesting that paternal influences may operate through indirect or context-dependent mechanisms rather than linear transmission alone.

From a clinical and preventive perspective, the findings indicated that interventions aimed at strengthening attachment security in adolescents may be effective in reducing the risk of NSSI. Family-based intervention approaches focused on enhancing parent–child relationships and reinforcing adolescents’ perceptions of emotional support may indirectly decrease the likelihood of self-injurious behaviors. Furthermore, tailoring these interventions to address the distinct contributions of insecure attachment in fathers and mothers may provide a more comprehensive strategy for improving mental health outcomes among adolescents [15, 36–38].

These conclusions expanded upon existing literature examining associations among NSSI, attachment patterns, and family-related processes. Whereas prior studies documented the associations between insecure attachment and self-injurious behavior [9, 13–15], the present investigation clarified the pathway through which parental insecure attachment was associated with NSSI via insecure attachment in adolescents. Furthermore, the findings highlighted the contribution of paternal attachment patterns along with maternal influences, consistent with recent evidence emphasizing the significant role of fathers in adolescent psychosocial outcomes [39–42].

Several limitations should be acknowledged. First, the cross-sectional design restricted the ability to draw causal inferences and precludes determination of the temporal direction of the observed associations. Moreover, bidirectional influences between adolescents and parents cannot be excluded, particularly during adolescence, when adolescents’ behaviors may also shape parental emotional responses and attachment patterns. Second, the relatively small sample size (74 parent–adolescent triads) may limit the stability of parameter estimates and reduce statistical power in structural equation modeling. Caution is warranted in interpreting the magnitude of several relatively large path coefficients and marginal p-values. Third, the exclusive use of self-report instruments may have introduced social desirability bias and recall inaccuracies, which could affect the precision of the assessed constructs. Fourth, psychiatric diagnoses and NSSI history were determined through routine clinical assessment and medical record review rather than structured diagnostic interviews, which may limit diagnostic standardization across participants. Fifth, attachment in adolescents was assessed using an adapted version of the ECR, in which references to “partner” were replaced with “parent.” Although this approach has been used in previous attachment research, its psychometric properties have not been fully established in Chinese adolescent clinical populations, which may limit measurement precision. Additionally, several important psychosocial and clinical variables, including childhood trauma, parenting style, and personality functioning, were not assessed in the present study. Finally, the relatively small sample size and the specific characteristics of the study population may have limited the generalizability of the findings.

Future research would benefit from larger and more diverse samples, the use of multi-informant and observational assessment methods, and the examination of additional factors such as peer relationships, cultural context, childhood trauma, personality functioning, and biological markers that may mediate or moderate the association between parental attachment insecurity and NSSI [43–46]. Longitudinal designs are also needed to clarify the temporal and potentially bidirectional mechanisms underlying intergenerational transmission of attachment insecurity and its relationship with NSSI. Furthermore, evaluation of the effectiveness of interventions targeting parent–child attachment process and reducing the risk of NSSI represents an important direction for translational research.

## Conclusion

In summary, the findings highlighted the central mediating role of insecure attachment in adolescents in the association between parental insecure attachment and NSSI. The indirect influence of parental attachment patterns on self-injurious behavior during adolescence underscored the importance of addressing attachment security in adolescents within both preventive and therapeutic frameworks. By strengthening attachment security in adolescents and addressing the distinct contributions of insecure attachment in fathers and mothers, clinicians and researchers may be better positioned to develop targeted, family-centered interventions aimed at reducing the risk of NSSI and supporting healthier developmental trajectories during adolescence.

## Data Availability

The datasets used and/or analysed during the current study available from the corresponding author on reasonable request.

## Abbreviations

NSSI: Non-suicidal self-injury
SEM: Structural equation modeling
ECR: Experiences in Close Relationships Inventory
DSM-5: Diagnostic and Statistical Manual of Mental Disorders, Fifth Edition
HC: Healthy controls
MD: Mood disorder
ANOVA: Analysis of variance
CI: Confidence interval
OR: Odds ratio
SD: Standard deviation
WLSMV: Weighted least squares mean and variance adjusted
